# Transient Electromagnetic Processes Analysis in High Voltage Transmission Lines during Two-Phase Short Circuits

**DOI:** 10.3390/s23010298

**Published:** 2022-12-27

**Authors:** Tomasz Perzyński, Vitaliy Levoniuk, Radosław Figura

**Affiliations:** 1 Faculty of Transport, Electrical Engineering and Computer Science, University of Technology and Humanities, 26-600 Radom, Poland; 2 Department of Electrical Systems, Lviv National Agrarian University, 80381 Dubliany, Ukraine

**Keywords:** transient electromagnetic processes, long power line, mathematical simulation, Hamilton–Ostrogradskii principle, boundary conditions, short circuit, electrical network

## Abstract

The mathematical model of a fragment of a high-voltage electric network is developed in this paper. The network consists of a long power line with distributed parameters and an equivalent three-phase active-inductive load. Neumann and Robin—Poincare boundary conditions were used to identify the boundary conditions for the long line equation. The parameter output voltage (voltage at the end of the line) is introduced into the paper for further universal use of the developed line model. On the basis of the developed mathematical model, the program code is written in the algorithmic language *Visual Fortran*. By means of it, oscillograms of transient electromagnetic processes of voltages and currents in the form of spatial, temporal and temporal-spatial distributions during remote two-phase short circuits in the transmission line of high voltage are obtained. Two transient electromagnetic processes are analyzed in the present work. The first one was analyzed during the switching on of the line to the normal mode of operation with the subsequent transition to the emergency mode. The second one was analyzed during the switching on the line in the mode of a remote two-phase short circuit to the ground. The results of transient electromagnetic process simulation in the form of analyzed figures are shown. All the results presented in this paper were obtained exclusively using numerical methods.

## 1. Introduction

It is crucial to take into account the matter of emergency modes when designing electrical networks. It is important to do as they are usually accompanied by damage to the elements of electrical networks. The most dangerous and common emergency mode is a short-circuit mode. After all, there are important short-circuit currents in the components of electrical networks. Thermal and electrodynamic actions are brought about by them and are also accompanied by a sharp voltage decline in the electrical grid.

Short-circuit currents can heat up conductive parts or even melt wires (temperatures can be as high as 20,000 K). Therefore, there comes a fragmentary or complete termination of the electricity supply to users. Additionally, such damage leads to the destruction caused by an electric arc that occurs at the point of the short circuit and may alter adjacent objects. The voltage is decreased by short circuits at the network nodes, which modify the steadiness processes disruption and the stability of the power system as well.

Relay protection and automation must be properly designed for electrical networks in order to avoid the above-mentioned consequences of short circuits. It is necessary to perform a comprehensive study of emergency mode parameters. This requires an analysis of transient electromagnetic processes in the appropriate operation modes. Two-phase short circuits are no exceptions.

The method of mathematical modeling is one of the most effective and optimal current ways of analyzing transient electromagnetic processes in electrical network elements. Talking about the mathematical modeling of transient electromagnetic processes in the elements of electrical networks, we can apply circular and field approaches.

Today, many researchers prefer circular approaches to the analysis of transient processes in electrical network elements. This approach is based on the equivalence of the well-known telegraph equation with a circular electric circuit of substitution. This means that from the field level, they descend to the circle level; that is, from the field setting of the problem, they move to the circle level, thus reducing a priori the degree of adequacy of the model of the line and therefore the degree of adequacy of the model of the entire object. It cannot be said that such an approach will be wrong, but it is clear that when it is used, the physical essence of the telegraph equation itself is lost.

The circular approach is considered to be a common tool that has already reached its limits in transient process modeling. Its application is based on the use of *RLC* substitution schemes.

Nowadays, there is a tendency to improve the methods and means of mathematical modeling of processes and systems. Thus, the reference publication of the International Council on Large Electrical Systems from February 2020 notes the need to use more advanced tools and methods for modeling power systems, including transient electromagnetic processes. The use of models that allow real-time evaluation of the studied parameters is especially relevant. Therefore, the field approach, in which the models are based on the theory of the electromagnetic field, is widely supported. This approach makes it possible to reproduce transient electromagnetic processes solely on the basis of the fundamental laws of applied physics.

Our research also includes ultra-high-voltage power lines. As is known [1,2,3], these lines have considerable lengths, which are commensurate with hundreds of kilometers. Therefore, changing operating modes have waved processes. For adequate reproducing of these processes, it is necessary to solve the long line differential equation (telegraph equation) [4,5].

However, there are some nuances. A long line differential equation is a partial differential derivative equation. It is known that in order to solve such equations, it is necessary to have boundary conditions. To find these boundary conditions, you need to use a mathematical model of the whole subsystem (both at the beginning and the end of the line), which, in the case of volumetric subsystems, creates a rather difficult task. The problem is that they are usually unknown or vaguely given. It is advised to use the boundary conditions of the second and third genera (Neumann and Robin—Poincare boundary conditions) to solve the long line differential equation.

Actually, in this paper, a technique of the mentioned boundary conditions identification to the long line differential equations is considered. On the one hand, this approach makes it possible to calculate transient electromagnetic processes with a high degree of adequacy. On the other hand, it is possible to use the model of a three-phase power line autonomously when modeling electrical networks that contain other elements.

## 2. Examination of a Recent Study

There are a large number of works devoted to the analysis of transient electromagnetic processes in power lines during short circuits in the scientific literature. Let us consider some of them close to the current work.

A mathematical model for the calculation of electromagnetic transient processes in three-phase lines has been developed in [6]. The basis of the developed model is differential equations with partial derivatives. They describe the electromagnetic state of the object under study. The boundary and initial conditions for the study of a wide range of practical problems are also displayed. The developed mathematical model is suitable for the calculation of transient electromagnetic processes in emergency modes in particular. In addition, the model can be used in two-phase short circuits as well.

This dissertation [7,8,9] shows a mathematical model of the transmission line that is based on the methods of lost elements and the space state of the line. The method that is introduced is an uncomplicated and practical procedure for modeling the three-phase transmission line directly in the time domain without explicit usage of inverse transforms. The line model considers the frequency-dependent parameters, taking into account the impact of the soil. In addition, it has been suggested in the paper to apply the procedure of analytical integration of equations of electromagnetic state, which makes it possible to study the transient and steady-state modes of line operation. The obtained results were verified in a satisfactory way and the performance of the *EMTP-RV* software suite has been presented.

In [10,11,12], a mathematical model of a power transmission line with classified parameters was created. It is based on the equations of a long line of the first order with a given boundary condition of voltage and current along the sides of the line.

In [13], the application of first-order long line equations for the study of transient processes in the ground wire is studied. The imitation is conducted in the frequency domain with the subsequent transition to time.

The paper [14] shows the mathematical model of a perfectly transposed three-phase power line. It is allowed by the model to calculate phase currents and voltages along the line as a function of the time coordinate. Currents and voltages are recorded in the form of equations of the state of the replacement electrical circuit. The phase and interphase parameters of the power line are taken into account. In this way, the mathematical model of the line is calculated in the software package *EMTP-RV*. The evaluation of the computer simulation’s results, which turns on the power line in the non-working stroke model, is introduced in the paperwork.

The three-phase power lines with distributed parameters pattern has been created in [15] with the usage of the software package *PSCAD*. This model can be used to imitate transient electromagnetic processes while switching short circuits and other modes of the line operator. The simulation of various modes is carried out by additional involvement of *R*, *L* and *C* elements. The traveling wave method was used to solve the long line equation.

The [16] presents the examination of overvoltages in the line of high voltage that was carried out. The transmission line in this case is represented by a number of connections of alternate electrical networks. The computer simulation was executed in the *ATP* software package.

In [17], surge simulations in the 500 kV line during a lightning strike were simulated with the support of the PSCD software package. The coronation phenomenon of wires was neglected during the simulation, and the running phase and interphase active conductivities were not taken into account.

For the analysis of transient processes in a three-phase power line with arbitrary voltage and current distributions in the line, it is suggested to use the numerical inverse Laplace transform algorithm in [18]. Here, the Laplace transform methodology is also applied to find voltages and currents along the line edges. Phase voltages and currents are obtained as logical functions of their frequency. It is indicated how the numerical inverse Laplace transform can be applied to gain the distribution of electromagnetic waves in a transmission line.

The transient electromagnetic processes in symmetric and asymmetric short circuits in different places of the high-voltage power line connected to the generating unit busbars are scrutinized in [19]. The equations of voltages and currents of the transmission line and substation buses are introduced in phase coordinates. It gives the possibility to model different asymmetric states easily. Here, a model for temporary electromagnetic process analysis has been developed in the *MatLab/Simulink* software package.

The available literature’s analysis has shown that most studies of transient electromagnetic processes in power lines are conducted by replacing the equation of the long line (telegraph equation) with a circular equivalent [14,15,16,17], which is not always effective. Moreover, it is possible to say that insufficient attention is given to the mathematical modeling of these processes in long power lines at the field level. Indeed, this work has been underway for a long time. Commonly used approaches require well-defined boundary conditions for the long line equation [6,10,13,18], or they are burdened by analytical integration [7,8,9]. With regard to the *MatLab/Simulink* software package, the distributed parameter line model built into the *Simulink* library is made easy. This prototype does not take into consideration the running resistance, phase and interfacial conductivity in order to simplify the calculations by the method of D’Alembert [19,20,21]. The same approaches are used in other software tools [22]. However, this may result in inaccuracy of the results.

Hence, the aim of the work is to improve methods of mathematical modeling and analysis of transient electromagnetic processes in long three-phase power lines in emergency modes.

## 3. Presentation of Basic Material

There are usually two main approaches to obtaining equations for the electromagnetic state of the studied objects. The first is a classical approach derived from the law of energy conservation. The second one is a variational approach based on minimizing the functionality of the examined object. Each of these approaches has its disadvantages and advantages, but when used correctly, they both lead to accurate results [23].

It is advised to use a modified Hamilton–Ostrogradsky principle (variational approach) for the analysis of transient electromagnetic processes in the elements of electrical networks [24]. This viewpoint avoids the decomposition of a single dynamic system. The first equations of the object’s electromagnetic state under study can be obtained purely on the basis of a single energy approach by constructing an extended Lagrange function [24]. This method is especially relevant for systems with distributed parameters, particularly for long power lines.

This thesis of transient electromagnetic process analysis in long power lines based on variational approaches in a single-line version was extended in [25] and further developed in [25]. To entirely duplicate these processes in long power lines, which often operate in asymmetric modes, they must be modeled in multiphase execution. On this account, there will be built a mathematical model of the line in three-phase execution.

**Figure 1:**
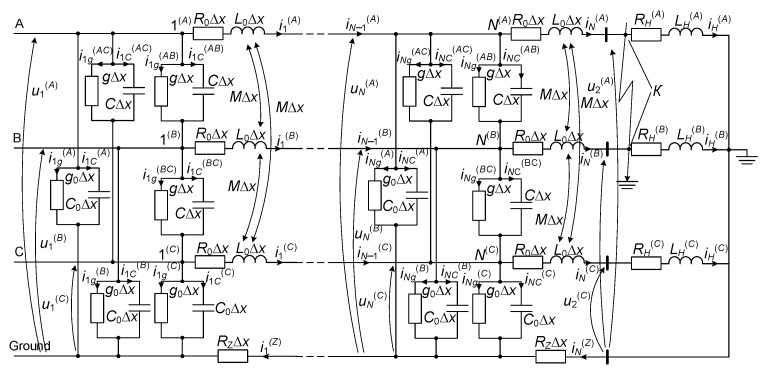
Calculation scheme of the studied fragment of the electrical network (for the first and last discrete sections of the line).

Figure 1 shows the calculation scheme of the electrical network’s fragment we are studying. The crucial element is a long power line. It is depicted in a three-phase design as a line with distributed parameters (here are shown only the first and last discrete nodes of the line). A voltage is applied to the beginning of the line. In the end, an equivalent three-phase active-inductive load is connected to it.

### 3.1. Hamilton–Ostrogradsky Principle

We have already mentioned that when building mathematical models of components of electrical networks and systems, we use the Hamilton–Ostrogradsky principle. This principle [24] extends the classical Lagrangian principle by adding two more components: the energy of dissipative forces in the system and the energy of non-potential forces acting on the object from the outside. Therefore, four types of energy act on the research object. Since we are considering an object with distributed parameters, we also use the concept of linear energy density. Next, based on the Hamilton–Ostrogradsky principle, an extended action functional is formed, after which it is minimized. As a result, the extremum of the action functional is obtained, which can be interpreted as a solution of the Euler–Lagrange equation for the subsystem with lumped parameters and the Euler–Poisson equation for the subsystem with distributed parameters. These solutions represent the mathematical model of the object under study. There are elements with both the concentrated and the distributed parameters in the fragment of an electric network we investigated. Therefore, the Hamilton–Ostrogradsky action functional looks like this [24]:

(1) $$S=\int_{0}^{t_{1}}L^{*}dt+\int_{0}^{t_{1}}\int_{l}^{}L_{l}dldt,\;\;\;I=\int_{l}L_{l}dl,$$

where *S*—action according to Hamilton–Ostrogradsky; *I*—energy functional; L_*_—extended Lagrange function, *L_l_*—linear density of the modified Lagrange function [25]:

(2) $$L^{*}={\tilde{T}}^{*}-P^{*}+\Phi^{*}-D^{*},\;\;\;\;\;\;\;\;L_{l}={\tilde{T}}_{l}-P_{l}+\Phi_{l}-D_{l},$$

where ${\tilde{T}}^{*}$—kinetic coenergy, P_*_—potential energy, Φ_*_—energy dissipation, and D_*_—energy of outside nonpotential forces, with index l being the corresponding linear densities of energies.

You can get a detailed look at the derivation of the equation of a long line with distributed parameters by our scientific association in the work [26] and other elements of electrical networks in the works [27,28]. Therefore, in order to reduce the volume of material, we propose ready-made equations for the studied fragment of the electrical network (Figure 1).

### 3.2. Mathematical Model of a Fragment of the Electric Network

The final equations of the electromagnetic state of the studied fragment of the electrical network in matrix-vector form are presented:

(3) $$\frac{\partial^{2}u}{\partial t^{2}}={\left(L_{0}{С}_{0}\right)}^{-1}\left(\frac{\partial^{2}u}{\partial x^{2}}-\left(L_{0}G_{0}+R_{0}{С}_{0}\right)\frac{\partial u}{\partial t}-R_{0}G_{0}u\right);$$

(4) $$\frac{di_{H}}{dt}=L_{Н}^{-1}\left(u_{2}-R_{H}i_{H}\right),$$

where

(5) $$L_{0}=\begin{array}{ccc} L_{0} & M & M\\ M & L_{0} & M\\ M & M & L_{0} \end{array},\;R_{0}=\begin{array}{ccc} R_{0}+R_{Z} & R_{Z} & R_{Z}\\ R_{Z} & R_{0}+R_{Z} & R_{Z}\\ R_{Z} & R_{Z} & R_{0}+R_{Z} \end{array};$$

(6) $${С}_{0}\;=\begin{array}{ccc} {С}_{0}+2С & -С & -С\\ -С & {С}_{0}+2С & -С\\ -С & -С & {С}_{0}+2С \end{array},\;G_{0}=\begin{array}{ccc} g_{0}+2g & -g & -g\\ -g & g_{0}+2g & -g\\ -g & -g & g_{0}+2g \end{array};$$

(7) $$R_{H}=\mathrm{diag}\left(R_{H}^{\left(A\right)},\;R_{H}^{\left(B\right)},\;R_{H}^{\left(C\right)}\right),\;L_{H}=\mathrm{diag}\left(L_{H}^{\left(A\right)},\;L_{H}^{\left(B\right)},\;L_{H}^{\left(C\right)}\right).$$

In Equations (5)–(7) *R*_0_, *g*_0_, *C*_0_, *L*_0_—resistance, conductivity, capacitance and inductance per unit length of the line, respectively; *g*, *C*—phase-to-phase conductivity and capacitance per unit length, respectively; *М*—mutual inductance per unit length; *R_Z_*—earth resistance per unit length; *R_H_*^(*k*)^, *L_H_*^(*k*)^—resistance and inductance of the corresponding phase of the equivalent load; *k* = *A*, *B*, *C*—the phase name.

The judgments presented by us require, during the study of transient processes in power lines, the solution of differential equations with partial derivatives (equations of a long line). Today, solving these equations is not a problem. Here, you can use such methods as the traveling wave method (D’Alembert’s method), the method of separation of variables, the method of straight lines, etc. Actually, the problem is finding the boundary conditions for the equation of a long line. It is known that there are boundary conditions of the first, second and third orders. In modern literature, boundary conditions of the first kind are often used to solve the equation of a long line. The use of boundary conditions of the first kind will be appropriate when the boundary conditions at the beginning and end of the line are known (currents, voltages, charges are given functions); on the other hand, in real problems of applied electrical engineering, the mentioned functional dependences of the boundary conditions are not always known, especially when it concerns the analysis of complex electrical networks that are connected by a long line. In this case, it is known that the voltage at the beginning of the line (**u**_1_ = **u**_│*х* = 0_), but not at the end of it. Ergo, it is necessary to locate only the boundary condition at the end of the line. Note that the line is loaded with an equivalent three-phase active-inductive load. We see that it is not possible to use this method in full. Therefore, we propose using boundary conditions of the second and third kinds. This approach makes it possible to solve the equation of a long line in the absence of the boundary stress function using an equation relative to its spatial derivative. The application of boundary conditions of the second and third kinds makes it possible to exclude the boundary stress from the system of differential equations in such a way that the sought stress is taken into account consistently during the integration of the latter. Thus, everything is connected in one system of equations.

In [25] symmetrical modes were considered for homogeneous long power lines. This is why the line was formed in a single-phase (single-line) design. It is suggested to use the boundary conditions of the second and third genera (boundary Neumann and Robin–Poincare conditions). Moreover, the equation can be obtained by Kirchhoff’s second law for electric circuits with distributed parameters. It is proposed to use this technique for three-phase systems. The mentioned equation in matrix-vector form is written below:

(8) $$-\frac{\partial u}{\partial x}=R_{0}i+L_{0}\frac{\partial i}{\partial t}.$$

By discretizing Equations (1) and (6) with the method of lines, using the notion of the central derivative, we obtain:

(9) $$\frac{dv_{j}}{dt}={\left(L_{0}C_{0}\right)}^{-1}\left(\frac{1}{{\left(\Delta x\right)}^{2}}\left(u_{j-1}-2u_{j}+u_{j+1}\right)-\left(L_{0}G_{0}\right.\right.+\left.R_{0}{С}_{0}\right)v_{j}-R_{0}G_{0}\left.u_{j}\overset{}{\underset{}{}}\right)\;,\;\frac{du_{j}}{dt}=v_{j};$$

(10) $$\frac{di_{j}}{dt}=L_{0}{}^{-1}\frac{1}{2\Delta x}\left(u_{j-1}-u_{j+1}\right)-L_{0}{}^{-1}R_{0}i_{j},\;j=2,\ldots ,N.$$

Write Equations (9) and (10) for the last discrete node of the line (*j = N*):

(11) $$\frac{dv_{N}}{dt}={\left(L_{0}C_{0}\right)}^{-1}\left(\frac{1}{{\left(\Delta x\right)}^{2}}\left(u_{N-1}-2u_{N}+u_{N+1}\right)-\;\left(L_{0}G_{0}\right.\right.+\left.R_{0}{С}_{0}\right)v_{N}-\;\;R_{0}G_{0}\left.u_{N}\overset{}{\underset{}{}}\right),\;\frac{du_{N}}{dt}=v_{N};$$

(12) $$\frac{di_{N}}{dt}=L_{0}{}^{-1}\frac{1}{2\Delta x}\left(u_{N-1}-u_{N+1}\right)\;\;-L_{0}{}^{-1}R_{0}i_{N}.$$

Analyzing Equations (11) and (12), we see that they have an unknown voltage in the fictitious node **u***_N_*_+1_. This makes it impossible to find the voltage at the last discrete node of line **u***_N_* Equation (11). Additionally, we cannot find the current in the last discrete branch of line **і***_N_* Equation (12). The voltage **u***_N_*_+1_ does not exist in nature, and it has no physical meaning. It is a purely fictitious mathematical quantity.

The method of finding this voltage is described and tested in the work [29], however, it is not perfect, because in order to build mathematical models of electrical networks of various configurations of line connections with other elements, the fictitious voltage **u***_N_*_+1_ will also change, which additionally requires changes in line models. We propose to make the line model more autonomous (universal); for this, we will introduce the “output voltage” parameter into the mentioned model (voltage at the end of the line **u**_2_ = **u**_│*х = l*_ (**u***_N_* ≠ **u**_│*х = l*_), see Figure 1). Let us present the sequence of finding the fictitious voltage **u***_N_*_+1_ at the end of the power transmission line.

For the last discrete contour of the line (see Figure 1), we write the equation according to Kirchhoff’s second law:

(13) $$\frac{di_{N}}{dt}={\left[\Delta xL_{0}\right]}^{-1}\left(u_{N}-u_{2}-\Delta xR_{0}i_{N}\right)\;.$$

We equate Equation (12) (written for the last discrete node of the line (*j = N*)) with Equation (13).

(14) $$L_{0}{}^{-1}\frac{1}{2\Delta x}\left(u_{N-1}-u_{N+1}\right)-L_{0}{}^{-1}R_{0}i_{N}={\left[\Delta xL_{0}\right]}^{-1}\left(u_{N}-u_{2}-\Delta xR_{0}i_{N}\right).$$

Expressing from Equation (14) the voltage function of fictitious node **u***_N_*_+1_, we obtain:

(15) $$u_{N+1}=u_{N-1}+2\left(u_{2}-\;\;u_{N}\right),$$

It is possible to avoid the fictitious voltage change when changing the configuration of the power line connection scheme with other elements of the electrical network. For this, use expression (15) as a function of the fictitious voltage **u***_N_*_+1._ This makes the mathematical model of the line autonomous (universal). However, there is now a need to find the voltage **u**_2_, which appears in expression (15). This voltage changes depending on the network circuit configuration. Since it has no direct effect on the line model, the line model will not change. 

The current **i***_N_* is identically equal to the current **i***_H_* (see Figure 1). Therefore, taking into account the initial conditions [24], we can write (equate the derivatives of currents):

(16) $$i_{H}\equiv i_{N}\;\;\;\Rightarrow \;\;\frac{d}{dt}i_{H}\equiv \frac{d}{dt}i_{N}\;.$$

where, taking into account Equations (4) and (13), we obtain:

(17) $$L_{H}{}^{-1}\left(u_{2}-R_{H}i_{N}\right)={\left[\Delta xL_{0}\right]}^{-1}\left(u_{N}-u_{2}-\Delta xR_{0}i_{N}\right)\;.$$

Expressing from Equation (17) the voltage function **u***_2_*, we obtain:

(18) $$u_{2}={\left[L_{Н}^{-1}+{\left[\Delta xL_{0}\right]}^{-1}\right]}^{-1}\left[{\left[\Delta xL_{0}\right]}^{-1}\left(u_{N}-\;\;\Delta xr_{0}i_{N}\right)+L_{Н}^{-1}r_{Н}i_{Н}\right].$$

The value of the current in the first discrete branch of the line or, if necessary, in all discrete line branches, can be calculated by discretizing Equation (8) by the lines method, but now using the concept of the right derivative:

(19) $$\frac{di_{j}}{dt}=L_{0}^{-1}\frac{1}{\Delta x}\left(u_{j}-u_{j+1}\right)-L_{0}^{-1}R_{0}i_{j},\;\;\;\;j=1,\ldots ,N.$$

The system of differential Equations (4), (9), (10) and (15) is subject to joint integration, noting Equations (5)–(7), (15), and (18).

## 4. Computer Simulation Outcome

The program code in the algorithmic programming language *Visual Fortran* was written to perform a computer simulation based on the developed mathematical formula. The code reproduces transient electromagnetic processes in the studied fragment of the electrical network (Figure 1).

### 4.1. Description of the Order and Parameters of the Simulation

A computer simulation was performed to study transient electromagnetic processes. They occur in the electrical network fragment presented in Figure 1. The simulation was performed for two experiments in which remote two-phase short circuits to the ground were investigated (short circuits at the end of the power line) (point *K*, see Figure 1).

The simulation was carried out as follows in the first experiment. The transmission line was turned on at time *t* = 0 s, with an asymmetric equivalent three-phase active-inductive load on the normal mode of operation. The specifics of phase-controlled switching (the switch is not modeled in the current work) were taken into consideration; notably, the line was switched on so that the phase voltage functions start from zero. Phase *A* was turned on at time *t* = 0 s, phase *В* − *t* = 0.00333 s, phase *С* − *t* = 0.00666 s. A two-phase short circuit to ground was imitated after entering the steady condition, at time *t* = 0.11 s, at the end of the power line (phases *A* and *B*, see Figure 1).

The second experiment showed the power line that was switched on for a two-phase short circuit at the end of the power line at time *t* = 0 s (as in the previous experiment, the short circuit to the ground was simulated in phases *A* and *B*).

The operation of emergency automation and relay line protection during the computer simulations was not taken into account. That is why the simulation results are presented without switching off short-circuit currents. It is obvious that this option is not entirely real; however, the simulation of switching processes of short-circuit currents is not in the scientific interests of the current work.

The parameters of the real power transmission line 750 kV, which connects the substation “Zakhidnoukrainska” (Ukraine) with the substation “Albertirsha” (Hungary) with a length *l* = 476 km, were accepted for research.

The parameters of the long line with distributed constants are as follows: *R*_0_ = 1.9 × 10^−5^ Om/m, *L*_0_ = 1.665 × 10^−6^ H/m, *С*_0_ = 1.0131 × 10^−11^ F/m, *g*_0_ = 3.25 × 10^−11^ Sm/m, *С* = 1.0122 × 10^−12^ F/m, *g* = 3.25 × 10^−13^ Sm/m, *R*_Z_ = 5 × 10^−5^ Om/m, *M* = 7.41 × 10^−7^ H/m. The parameters of the equivalent three-phase active-inductive load are as follows: *R_Н_*^(*А*)^ = 420 Om, *R_Н_*^(*В*)^ = 380 Om, *R_Н_*^(*С*)^ = 400 Om, *L_Н_*^(*А*)^ = 0.85 H, *L_Н_*^(*В*)^ = 0.7 H, *L_Н_*^(*С*)^ = 0.75 H. Computer simulation was performed with the following mode parameters: *u*_1_^(*A*)^ = 632 sin(ω*t*) kV, *u*_1_^(*B*)^ = 632 sin(ω*t* − 120º) kV, *u*_1_^(*C*)^ = 632 sin(*ωt* + 120º) kV. The step of spatial discretization of differential equations with partial derivatives by the method of lines is equal to: Δ*х* = *l*/20 = 476/20 = 23.8 km Conventional differential equations were integrated by the 6th order Gir method with a step of Δ*t* = 27 μs.

Since we analyze three-phase states, all the distributions of voltage and current functions considered in the article are denoted as follows: phase *A*—yellow lines, phase *B*—green, and phase *C*—red.

### 4.2. Experiment No. 1

**Figure 2:**
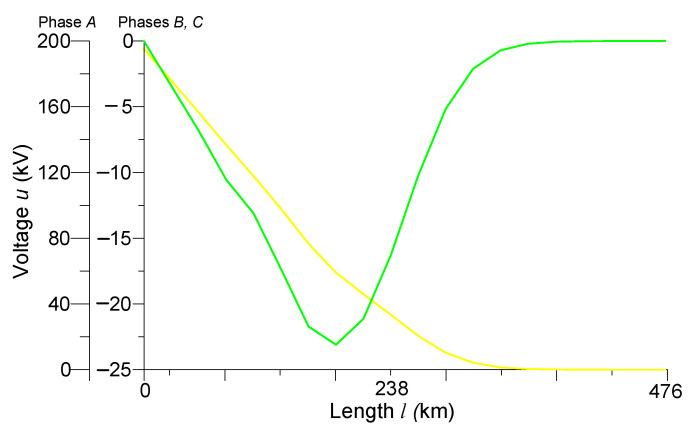
Spatial distributions of phase voltages in the line at time *t* = 0.001 s.

**Figure 3:**
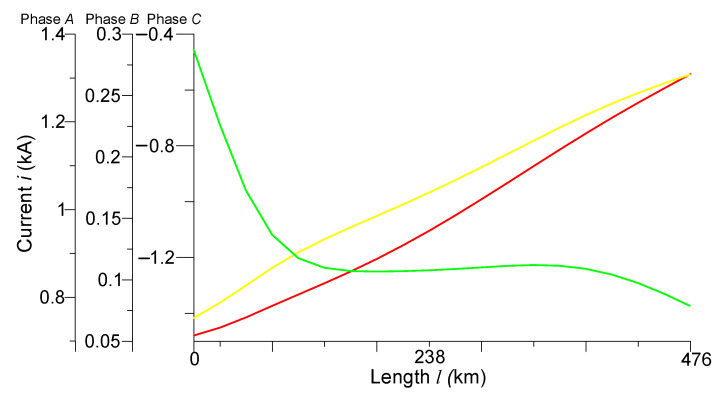
Spatial distributions of phase currents in the line at time *t* = 0.007 s.

Figure 2 and Figure 3 present the spatial distributions of phase voltages at time *t* = 0.001 s and phase currents at time *t* = 0.007 s, respectively. These figures very well reflect the course of wave electromagnetic processes in the power line, so they will be analyzed.

From Figure 2 it can be seen that during the beginning of the transmission line to normal operation at time *t* = 0.001 s, the voltage of phase *A* at the beginning of the line has a value of 200 kV. It was observed that in the time of 0.001 s, the electromagnetic wave had not yet reached the end of the line. At a distance of 350 km from the beginning of the line, the voltage was still zero. Phases *B* and *C* have not yet been switched on, so the green and red lines overlap. However, voltages are present on them (in the middle of the 22 kV line). This is because the line model takes into account the inter-inductive relationships between the phases, so the phenomenon of electromagnetic induction can be noticed.

We can see from Figure 3 that the current of phase *A* at the beginning of the line has a value of 0.75 kA. It is linearly increasing along the line to a value of 1.3 kA. The current of phase *B* at the beginning of the line has a value of 0.27 kA, in the middle of the line—0.12 kA, and at the end—0.07 kA. The current of phase *C* at the beginning of the line has a value of −1.45 kA and it is decreasing linearly along the line to a value of –0.5 kA.

**Figure 4:**
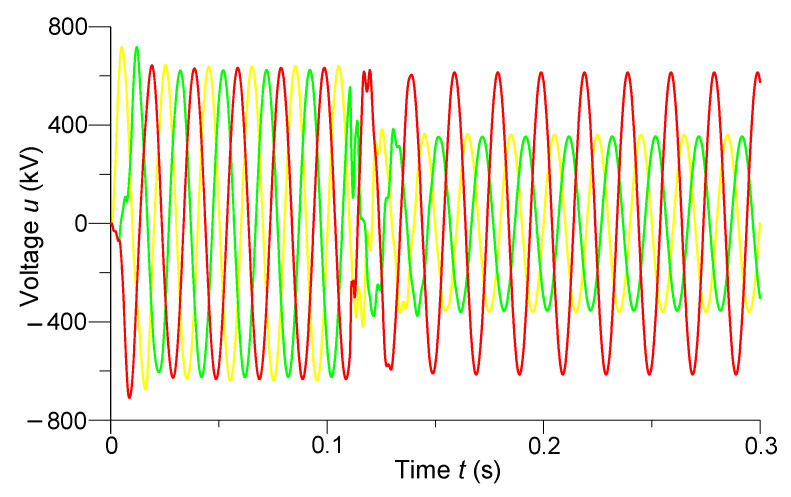
Transient processes of phase voltages in the middle of the line.

**Figure 5:**
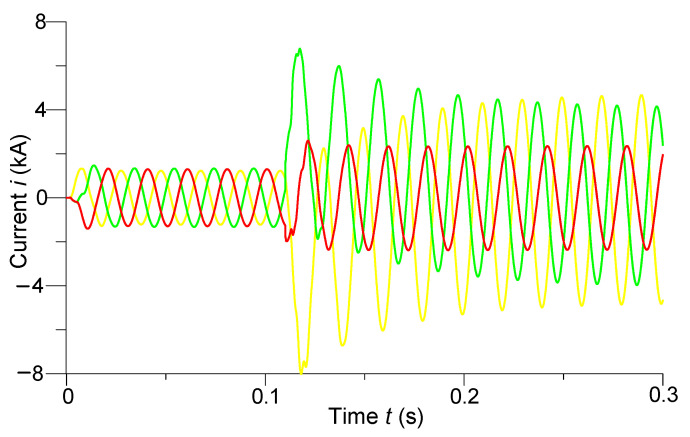
Transient processes of phase currents at the end of the line.

In Figure 4 and Figure 5 are shown the transient processes of phase voltages in the middle of the line and phase currents at the end of the line, respectively.

We can see from analyzing the voltage transient processes (Figure 4) that despite the use of controlled switching, overvoltages are still present when the line is turned on. For example, the phase instantaneous amplitude values of the overvoltages of phases *A* and *B* reached 717 kV, which was 1.11*U_MW_*. The instantaneous voltage amplitude values were approximately 620 kV after entering the steady state. The steady-state amplitude values of the voltages of phases *A* and *B* decreased to 352 kV after the occurrence of a two-phase short circuit at the end of the line. The voltage of phase *C* in the middle of the line did not change in the mentioned short circuit.

We observed a completely different situation with the transient processes of phase currents when the line was turned on. Here, controlled switching has affected the shock currents—they are virtually absent. We can see that the currents acquired instantaneous amplitude values of approximately 1.33 kA after entering the steady state. The shock current of phase *A* reached the value of −8 kA, and phase *B*—6.78 kA after a short circuit. There is practically no shock current in phase *C*. Only after a short circuit did the steady-state amplitude value of the current of the undamaged phase increase to 2.34 kA.

**Figure 6:**
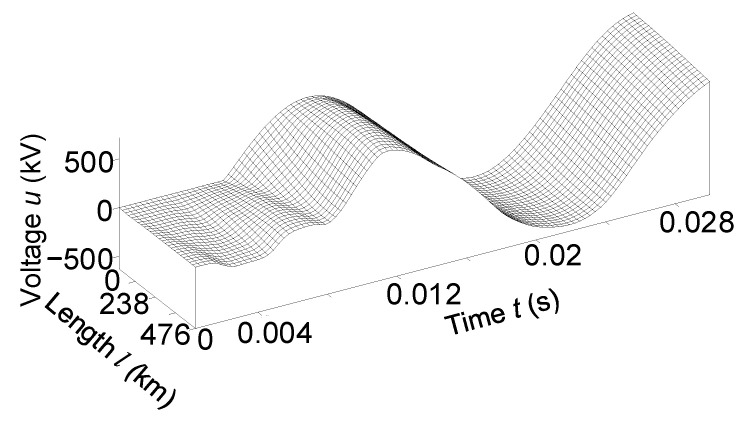
Temporal-spatial voltage distribution of phase *B* in line at the time *t* ∈ (0; 0.03) s.

**Figure 7:**
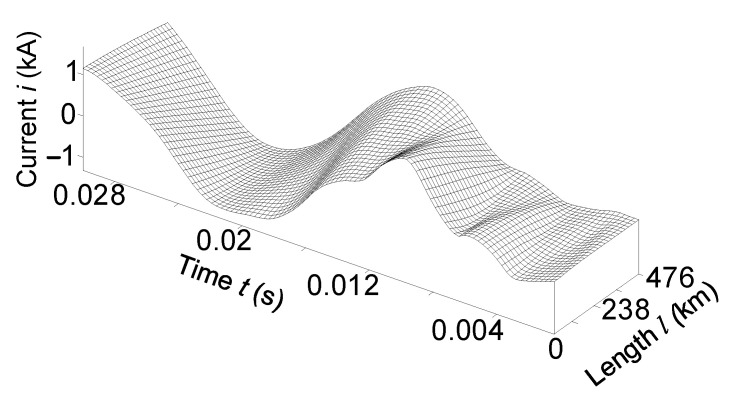
Temporal-spatial current distribution of phase *B* in line at the time *t* ∈ (0; 0.03) s.

Figure 6 and Figure 7 show the temporal-space voltage and current distributions of phase *B*. These figures are made in *3D* format; they combine both the time and space distributions of voltage and current in a line. These figures show how electromagnetic waves move in space along the transmission line.

Analysis of the mentioned figures shows that when the line is switched on for an equivalent asymmetric active-inductive load. The phase *B* voltage had the largest fluctuations at the end of the power line (Figure 6). Phase *B* current—at the beginning (Figure 7).

It is advisable to analyze Figure 6 together with Figure 2 and Figure 4, and Figure 7 with Figure 3 and Figure 5.

### 4.3. Experiment No. 2

**Figure 8:**
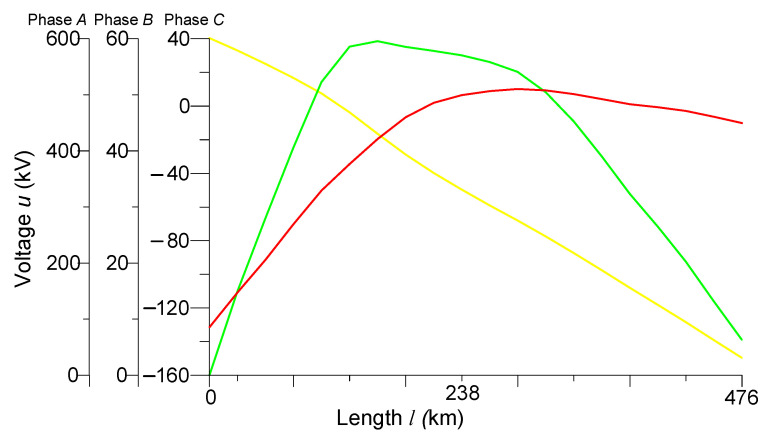
Spatial distributions of phase voltages in the line at time t = 0.004 s.

**Figure 9:**
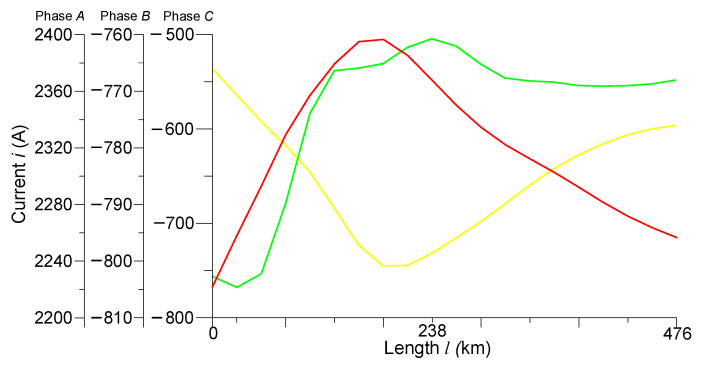
Spatial distributions of phase currents in the line at time *t* = 0.004 s.

Figure 8 and Figure 9 show the spatial distributions of phase voltages and phase currents at time *t* = 0.004 s, respectively.

Analyzing the spatial distributions of phase voltages at time *t* = 0.004 s (Figure 8), we see that the voltage of phase *A* had a value of 600 kV at the beginning of the line. It decreased linearly along the line to zero. This is due to the fact that the line was switched on in the mode of a two-phase short circuit to the ground at the end of the transmission line (phases *A* and *B*). As for the voltage of phase *B*, it had a zero value at the beginning of the line, 60 kV in the middle of the line, and at the end of the line. The same as the voltage of phase *A*, it was also equal to zero. The voltage of phase *C* at the beginning of the line had a value of −130 kV, decreasing in the middle of the line to zero, and at the end of the line again increased to −10 kV. Since at time *t* = 0.004 s, phase *C* was not yet turned on, it can be concluded that the presence of voltage in this phase was caused by the phenomenon of electromagnetic induction.

Figure 9 shows that the spatial distributions of the phase currents in the line have nonlinear characters. The current of phase *A* at the beginning of the line was 2380 A, decreasing in the middle of the line to 2240 A, and at the end of the line, increasing again to 2340 A. The spatial distributions of the currents of phases *B* and *C* were somewhat similar because after switching on phase *B* (0.0007 s), phase *C* was generally turned off. That is why no significant changes had yet occurred. The current of phase *B* had a value of −805 A and phase *C* −760 A at the beginning of the line. The current of phase *B* had a value of −760 A and phase *C* −500 A in the middle of the line. The current of phase *B* had a value of −770 A, and phase *C* −720 A at the end of the line.

**Figure 10:**
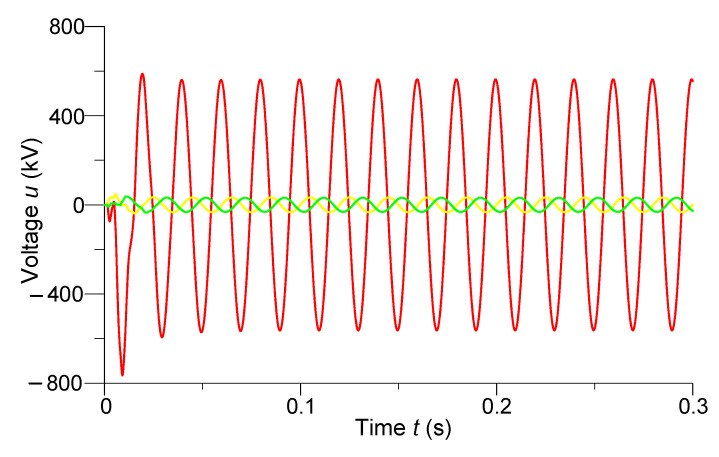
Transient processes of phase voltages on the last discrete node of the line (24 km to the end of the line).

**Figure 11:**
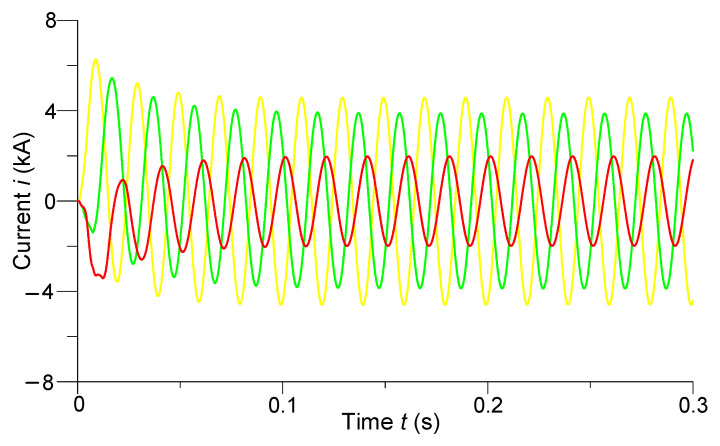
Transient processes of phase currents in the middle of the line.

Figure 10 and Figure 11 show the transients of phase voltages at the last (*N*-th) discrete node of the line (24 km to the end of the line) and phase currents in the middle of the line, respectively.

In Figure 10, we can see that when the power line was switched on in the two-phase short circuit to the ground at the end of the line, the voltages of the damaged phases *A* and *B* had instantaneous amplitude steady-state values of 33 kV. Additionally, we see that when the line was turned on in the mentioned emergency mode on the undamaged phase *C,* there was an overvoltage of 763 kV, which was 1.18*U_MW_*. After the transition process, the voltage of phase *C* at the last discrete node of the line acquired a steady-state amplitude value of 572 kV.

Making an analysis of Figure 11, we can see that when the line was switched on for a two-phase short circuit at the end of the line, there were shock currents in the middle of the line on phases *A* and *B* having values of 6.27 kA and 5.45 kA, respectively. The shock current of the undamaged phase had a value of 3.75 kA. There were significant aperiodic components in the currents of all phases. They attenuated after the transition process, and the phase currents in the middle of the line acquired the following steady-state amplitude values: phase *A* current—4.5 kA, phase *B*—3.9 kA, and phase *C*—2 kA.

**Figure 12:**
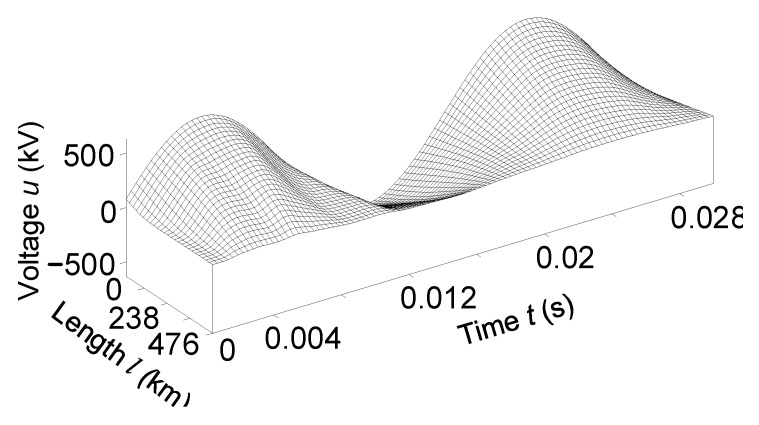
Temporal-spatial voltage distribution of phase *A* in line at the time *t* ∈ (0; 0.03) s.

**Figure 13:**
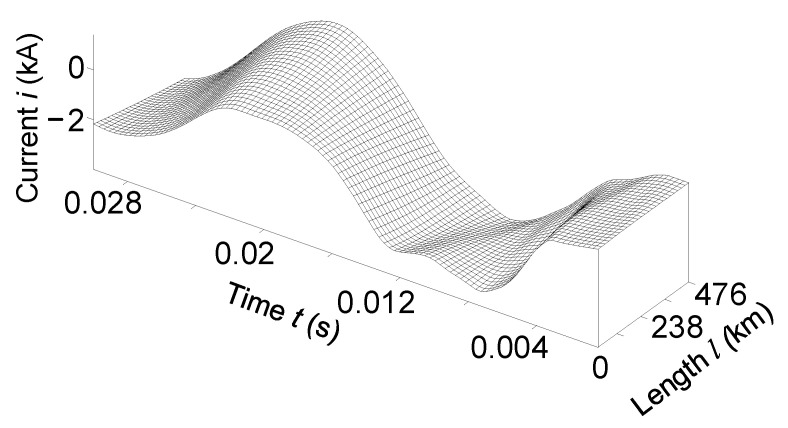
Temporal-spatial current distribution of phase *C* in line at the time *t* ∈ (0; 0.03) s.

In Figure 12 and Figure 13, we present the temporal-spatial distributions of phase *A* voltage and phase *C* current, respectively. It better displays the picture of transient electromagnetic processes in the transmission line when it is turned on in the remote two-phase short circuit to the ground.

It is advisable to analyze Figure 12 together with Figure 8 and Figure 10, and Figure 13 with Figure 9 and Figure 11.

## 5. Conclusions

Neumann and Robin Poincare’s conclusion of boundary conditions to identify boundary conditions to the differential equation of a long line of the second order makes it possible to effectively sort the problems out related to the analysis of transient electromagnetic processes in high-voltage lines, where they have to be considered as distributed parameters.

The introduction to the mathematical model of the line is based on a discretized equation of a long line. To solve this problem, the boundary conditions of the second and third genera are applied; the parameter output voltage (voltage at the end of line u2) allows the line model to be more autonomous and universal on the one hand. On the other hand, pens offer wider possibilities for reproduction of emergency states of operation of the line.

A comparative analysis of the transient electromagnetic processes of remote two-phase short circuits to the ground showed that the shock currents of the short circuit that occurred after the system entered the steady state (experiment No. 1) were 25% greater than when the line was turned on for a similar short circuit (experiment No. 2). After analyzing the overvoltages of the undamaged phase, we can see that when the line was started for a two-phase short circuit (experiment No. 2), they were 10% higher than in the case of a short circuit that occurred after the steady state of operation (experiment No. 1).

Information about wave processes in the line in 3D format temporal-spatial distributions of voltages and currents is maximally illuminated. They also confirm the physical principles of electrodynamics regarding the flow of wave electromagnetic processes in long power lines. They indicate the high adequacy of the developed mathematical formula.

The content of this work will be used in further research on the joint operation of turbogenerators, unit transformers, switching facilities and ultra-high voltage long transmission lines.

## Data Availability

Data are contained within the article.
